# Clustering of electronic health records in atrial fibrillation patients and impact on prognosis and patient trajectories: a UK linked-dataset study

**DOI:** 10.1093/ehjdh/ztaf032

**Published:** 2025-04-05

**Authors:** Zengqi Zhang, Hiroyuki Yoshimura, Dionisio Acosta-Mena, Carina Teixeira, Chris Finan, Gregory Y H Lip, A Floriaan Schmidt, Rui Providencia

**Affiliations:** Institute of Health Informatics Research, University College London, 222 Euston Road, London NW1 2DA, UK; Institute of Health Informatics Research, University College London, 222 Euston Road, London NW1 2DA, UK; Cegedim Rx Ltd, Second Floor, Building 2, Buckshaw Station Approach, Buckshaw Village, Chorley PR7 7NR, UK; Institute of Psychiatry, Psychology and Neuroscience, Kings College London, London, UK; Institute of Cardiovascular Science, University College London, London, UK; Liverpool Centre for Cardiovascular Science at University of Liverpool, Liverpool John Moores University and Liverpool Heart and Chest Hospital, Liverpool, UK; Danish Center for Health Services Research, Department of Clinical Medicine, Aalborg University, Aalborg, Denmark; Department of Cardiology, Lipidology and Internal Medicine with Intensive Coronary Care Unit, Medical University of Bialystok, Bialystok, Poland; Institute of Cardiovascular Science, University College London, London, UK; Institute of Health Informatics Research, University College London, 222 Euston Road, London NW1 2DA, UK; Barts Heart Centre, Barts Health NHS Trust, St. Bartholomews Hospital, West Smithfield, London EC1A 7BE, UK

**Keywords:** Atrial fibrillation, Depression, Anxiety, Electronic health records, Unsupervised Machine Learning

## Abstract

**Aims:**

Atrial fibrillation (AF) is characterized by heterogeneity in presentation, comorbidity profile and prognosis, with different AF subphenotypes having been previously suggested. Mental health disorders are common in the AF population. The current classification of AF, based on episode duration, fails to capture the complexity of the condition. Machine learning (ML) techniques and utilization of information on mental health disorders might improve identification of different and actionable AF subphenotypes.

**Methods and results:**

We utilized Nationwide UK data from the Clinical Practice Research Datalink (199 308 AF patients; age 75.4 ± 12.6; 49.2% women) and unsupervised ML for clustering (k-means). Twenty-five clinical features were used in the model, including the presence of mental health disorders (anxiety, depression, and psychosis). Outcomes were assessed at 5 years across different clusters. We identified five different clusters of AF patients with specific characteristics and behaviour. Clusters were labelled based on the most prevalent features: (i) elderly and cardiopaths; (ii) young age and mental health disease; (iii) elderly and hypertensive; (iv) middle age and depression; and (v) very elderly. Mental health disorders were present in 18% at baseline. When comparing across the different clusters, significant differences were observed for the rates of the different assessed outcomes: higher mortality, heart failure and dementia in cluster (v), cancer and anxiety or depression in cluster (iii).

**Conclusion:**

Using unsupervised clustering we identified five distinct clinically actionable AF subphenotypes. The differences in outcomes and event rates at 5 years, suggests the possibility of specific tailored therapy and interventions requiring further investigation. Management of mental health should be part of holistic or integrated care management in this population.

## Graphical Abstract

**Graphical Abstract:**
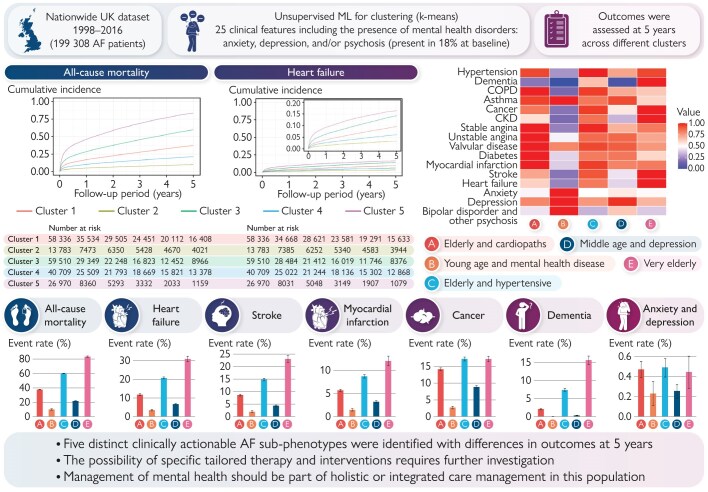

## Introduction

Atrial fibrillation (AF) is the most frequent cause of sustained arrhythmia in clinical practice, and it has been estimated that, in 2019, 59.7 million individuals lived with AF.^1^ AF often co-exists with multiple associated co-morbidities such as stroke, heart failure, coronary artery disease, and hypertension, which contribute to the hospitalization and prognosis of affected individuals.^2^

Besides being often described alongside cardiovascular disorders, an association between the AF and mental health disorders has been increasingly recognized.^3–5^ Indeed, recent guidelines highlighting depression as one of AF-related outcomes.^6^ This is all the more important given that mental health disorders contribute to 16% of disability Worldwide, and result in annual losses of >4.7 trillion USD.^7^ Depression and anxiety are two most prevalent mental health disorders, affecting 691 million individuals in 2021.^8^

AF is characterized by heterogeneity in presentation and comorbidity profile, with different AF subphenotypes having been previously suggested.^2^ The current taxonomy of AF, based on episode duration, fails to capture the complexity of the condition.^9^ An improved understanding of the different AF subphenotypes, with clarification of the role of mental health disorders within these groups might be of importance. Indeed, different AF subphenotypes may evolve differently, with different trajectories and complications, requiring more tailored treatment approaches. This knowledge and complex comorbidity profiles could potentially be elucidated through machine learning (ML) techniques like cluster analysis, as previously done for other cardiovascular and respiratory disorders.^10,11^

In this study, our aim was to utilize cluster analysis to identify clinically actionable subphenotypes of AF patients.

## Methods

The study protocol ‘*Natural History of Atrial Fibrillation in the United Kingdom’* was approved by the Medicines and Healthcare products Regulatory Agency Independent Scientific Advisory Committee (17_205). Data provided by the Clinical Practice Research Datalink (CPRD), include patients in the UK with linked data of primary care consultations, hospital data (Hospital Episodes Statistics), and death registry data (Office for National Statistics).^12,13^ These data are representative of the age, gender, and geographic distribution of the UK population^14^ and showed high quality and completeness of clinical information recorded.^12,13,15^

Our cohort was composed of individuals aged 18 years or older registered in the current primary care practice for at least one year. The study period was between 1 January 1998 and 31 May 2016, and individuals were excluded if they had a prior history of AF before study entry. The Read codes (for primary care) and International Classification of Diseases tenth revision (ICD-10) codes (for secondary care) of AF were retrieved from the Health Data Research UK Caliber phenotype library.^16^ AF was defined using ICD-10 code I48 and Read codes G573400, G573500, 3272.00, G573000, G573300, G573.00, and G573z00. Previous research has shown high validity and completeness of the disease definition in AF.^17^

Records with missing dates for AF diagnosis, prior history of AF before study entry, ‘historical’ or ‘inferred’ AF were excluded. A patient’s study entry date was defined as the latest of their CPRD entry date, up-to-standard GP date, or current registration date. A patient’s index date was considered as the AF diagnosis date. Follow-up ceased for the following reasons: death, the end date of registration with the practice, last day of the general practice data collection or the end of the study period (31 May 2016).

### Model input features—baseline covariates

A total of 25 clustering features were included and selected based on clinical expertise and previous publications,^18^ falling within different categories: demographics, lifestyle factors, co-morbidities and medication (see Supplementary material online, *Table S1*). Among the co-morbidities, 12 common chronic conditions associated with AF were included: hypertension, diabetes, valvular disease, unstable angina, stable angina, myocardial infarction, heart failure, stroke, cancer, chronic kidney disease, chronic obstructive pulmonary disease (COPD), asthma, and dementia. Common mental health disorders were also included. The proportion of individuals with a diagnosis recorded in their primary care or hospital admissions before or at the time of their initial diagnosis of AF was reported. Prescribed medication was defined as prescription of medications in the year before AF diagnosis and included medications from the British National Formulary chapter 2, covering cardiovascular disease.

### Clustering methods

Utilizing our AF population three clustering algorithms (K-means, Hierarchical clustering, K-medoids) were tested, and multiple sets of clusters were derived. After comparison and evaluation of these models, the best clustering methods were chosen for each dataset.

Subsets were generated using k-fold cross validation (15-folds), and one-fold was selected to represent the whole population in each dataset as previously reported.^19^ Basic principles and assumptions of each of the three unsupervised ML methods are outlined below.

K-means clustering is a method that identifies k number of clusters through iteratively minimizing the distance between points and their assigned cluster means. Principle component analysis was applied to the training dataset as a dimensionality reduction method prior to clustering. K-means clustering was performed using the *Hartigan-Wong* method with a maximum of 10 iterations and a single random initialization of cluster centers.^20^

Hierarchical clustering is a method of cluster analysis that builds a tree-like structure (dendrogram) to represent the relationships between data points. Dendrograms provide a clear visual representation of how data points are grouped and work well for data with complex and irregular shapes. Hierarchical clustering was performed using the complete linkage method, which defines the distance between two clusters as the most distant points in each cluster. This agglomerative (bottom–up) method starts with each data point as its own cluster and progressively merges the closest clusters based on the Euclidean distance.

K-medoids clustering, also known as partitioning around medoids, is a variant of K-means clustering that uses actual data points as cluster centres (medoids) instead of the average of data points. After selecting the medoids, each selected medoid and non-medoid point are swapped to minimize the sum of dissimilarities of all non-medoid points to their nearest medoid, and the algorithm stops when the sum no longer decreases. For k-medoids, the clustering algorithm is first applied to dataset after removing redundant rows due to limitations in the partitioning around medoids algorithm within R, and then assigns the clustering results to the whole dataset.

These three methods have previously been tested and performed satisfactorily in CPRD datasets.^19,21–23^

### Internal validation

After applying clustering methods on drop duplicated datasets separately, the silhouette coefficient and average silhouette width plot were used to measure how separated and distinct the clusters were. Duplicated rows were dropped for improving the effectiveness of clustering and speed up time. The silhouette coefficient was computed for each data subset to ensure the stability of the clustering method. The silhouette coefficient shows how strongly the assigned cluster is relative to the next closest cluster. A score closer to −1 or 1 indicates better cluster structure.^22^ Optimal number of clusters were applied with total within sum of square and Bayesian Information Criterion scores as well.

### Prognostic validation

First, survival probability was analysed comparing Kaplan–Meier 5-year survival plots. The survival plots for the first 5 years after the entry date were investigated to assess the trend of the lines clearly. The optimal number of clusters was obtained when the minimum overlap between all pairs of clusters was maximized (i.e. less overlap between clusters was better). Further validation was conducted by analysing the prevalence of co-morbidities, and incidence of AF-related outcomes (all-cause mortality, stroke, myocardial infarction, heart failure, dementia, and anxiety or depression), as defined on the recent European Society of Cardiology AF guidelines,^6^ in the first 5 years of follow-up. Univariate and multivariate Cox regression analysis assessed the association between AF-related outcomes and clusters. Multivariate Cox regression was adjusted for gender, age, life behaviours, and co-morbidities.

### Clustering analysis

The clusters were labelled after studying each cluster’s characteristics. Based on clinician input, a model, predicting cluster and survival using labels for identified clusters and clinically available factors, was developed and the patients with the same risk factor distributions were assigned with the same clusters. Relative prevalence of baseline characteristics was scaled to 100 for the subtype with highest prevalence of that factor and represented using a heat-map (warm/red representing relative prevalence closer to 1.0 and cold/blue closer to 0). The dominant co-morbidities or features of patients in a group were utilized for labelling a cluster.


$$\begin{array}{rl} & \mathrm{Relative}\;\mathrm{prevalence}\;\mathrm{rate}\;=\\ & \;\frac{\mathrm{Prevalence}\;\mathrm{rate}\;\mathrm{of}\;\mathrm{the}\;\mathrm{subtype}}{\mathrm{The}\;\mathrm{highest}\;\mathrm{prevalence}\;\mathrm{rate}\;\mathrm{of}\;\mathrm{the}\;\mathrm{subtype}\;\mathrm{in}\;\mathrm{each}\;\mathrm{cluster}} \end{array}$$

To illustrate the differences in time to events across clusters, cumulative incidence curves were utilized.

## Results

**Figure 1:**
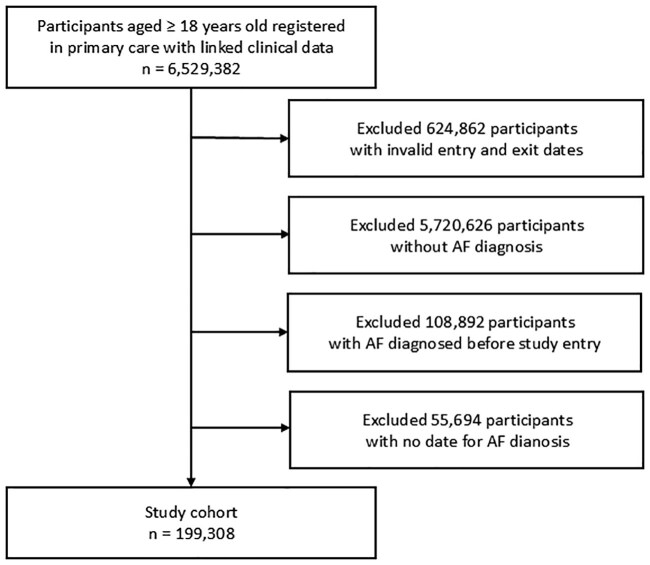
Study cohort selection.

After utilizing the pre-specified inclusion and exclusion criteria, out of a total of 6 529 382 patients, we identified 199 308 patients with new-onset AF following entry into the study (*Figure 1*).

All the variables were input to the clustering algorithm to ensure that no crucial information was omitted. Plot analysis to assess how the average silhouette width changed with number of clusters, suggested all selected ML methods showed potential in clustering the population (see Supplementary material online, Figure  *S1*). The possible number of clusters ranged from 2 to 5. The clusters obtained using the k-means and k-medoid algorithms had a higher average silhouette coefficient, fewer negative samples and a more balanced data distribution than hierarchical clustering (average silhouette width of about 4.5). Comparing the average silhouette width in other folds using different clustering algorithms, hierarchical clustering results tended to be unstable, i.e. comparing the maximum and minimum average silhouette width, the range of hierarchical clustering results was significantly different with regards to the clustering folds. Conversely, in the case of both k-means and k-medoids, after applying different clustering numbers to all other folds, the average silhouette widths showed adequate stability with regards to the original solution. Consequently, the optimal number of clusters was 4–5 and the selected ML methods were k-means or k-medoids (*Table 1*).

**Table 1:** Performance of three supervised ML models for predicting disease subtypes

		Silhouette, +			Silhouette, +	
Cohort	Method	Min	Max	Method	Min	Max
AF	K-means (*k* = 4)	0.471	0.493	K-means (*k* = 5)	0.441	0.469
	HC (*k* = 4)	0.283	0.52	HC (*k* = 5)	0.382	0.469
	k-medoids (*k* = 4)	0.452	0.478	PAM (*k* = 5)	0.43	0.461
	Best models	K-means/k-medoids		Best models	K-means/k-medoids	
	Best *n*:	4–5		Best *n*:	4–5	

HC, hierarchical clustering; PAM, partitioning around medoids.

On survival analysis, the optimal number of clusters with minimum overlap was five (see Supplementary material online, *Figure S2*). There was significant overlap between clusters for both k-means and k-medoids methods when *n* was 4. When cluster number was 5, k-means method showed less overlap between clusters than k-medoids, and therefore, k-means with 5 clusters, was applied.

The five clusters were compared based on demography, co-morbidities, risk factor burden, and medications. Age had an important role on cluster definition, but additional features with highest relative frequency in some clusters were utilized for labelling clusters as: (i) Cluster 1: elderly and cardiopaths; (ii) Cluster 2: young age and mental health disease; (iii) Cluster 3: elderly and hypertensive; (iv) Cluster 4: middle age and depression; and (v) Cluster 5: very elderly.

The proportion of patients assigned to each cluster was the following: 29.3% were assigned to elderly and cardiopaths, 6.9% to young age and mental health disease, 29.9% to elderly and hypertensive, 20.4% to middle age and depression, and 13.5% to the very elderly cluster (see Supplementary material online, Figure  *S3*). *Table 2* shows the demographics and prevalence of risk factors, co-morbidities and cardiovascular medication across the five clusters.

**Table 2:** Baseline characteristics of study population

AF	Total	Elderly and cardiopaths	Young age and mental health disease	Elderly and hypertensive	Middle age and depression	Very elderly
		1	2	3	4	5
Total (*n*)	199 308	58 336	13 783	59 510	40 709	26 970
%	100.00%	29.27%	6.92%	29.86%	20.43%	13.53%
Gender						
Male (*n* = 1)	101 323	31 783	9680	25 376	26 538	7946
%	50.84%	54.48%	70.23%	42.64%	65.19%	29.46%
Female (*n* = 0)	97 985	26 553	4103	34 134	14 171	19 024
%	49.16%	45.52%	29.77%	57.36%	34.81%	70.54%
Age Category						
18–50	8735	0	8735	0	0	0
%	0.00%	0.00%	63.38%	0.00%	0.00%	0.00%
50–70	45 757	0	5048	0	40 709	0
%	22.96%	0.00%	36.62%	0.00%	100.00%	0.00%
70+	144 816	58 336	0	59 510	0	26 970
%	72.66%	100.00%	0.00%	100.00%	0.00%	100.00%
Average age	75.5	74.9	44.7	83.8	63.5	92.2
(IQR)	17	4	11	4	7	4
Life behaviours						
Smoking	107 627	34 908	6891	30 154	25 598	10 076
%	54.00%	59.84%	50.00%	50.67%	62.88%	37.36%
Alcohol	75 048	23 824	4452	21.954	16.371	8447
%	37.65%	40.84%	32.30%	36.89%	40.21%	31.32%
BMI (mean)	27.72	28.13	30.54	26.14	30.04	24.28
(IQR)	7.30	6.90	9.40	6.40	8.00	6.00
Co-morbidities						
Hypertension	93 819	29 706	2016	32 232	16 131	13 734
%	47.07%	50.92%	14.63%	54.16%	39.63%	50.92%
Dementia	7461	1103	6	3687	140	2525
%	3.74%	1.89%	0.04%	6.20%	0.34%	9.36%
COPD	21 685	7691	306	7549	3854	2285
%	10.88%	13.18%	2.22%	12.69%	9.47%	8.47%
Asthma	25 847	8257	2050	7415	5679	2446
%	12.97%	14.15%	14.87%	12.46%	13.95%	9.07%
Cancer	43 289	13 306	743	15 618	6264	7358
%	21.72%	22.81%	5.39%	26.24%	15.39%	27.28%
CKD	32 930	8749	615	13 058	3580	6928
%	16.52%	15.00%	4.46%	21.94%	8.79%	25.69%
Stable angina	31 008	10 565	570	10 674	5131	4068
%	15.56%	18.11%	4.14%	17.94%	12.60%	15.08%
Unstable angina	11 545	3926	436	3466	2397	1320
%	5.79%	6.73%	3.16%	5.82%	5.89%	4.89%
Valvular disease	13 040	4169	790	3998	2612	1471
%	6.54%	7.15%	5.73%	6.72%	6.42%	5.45%
Diabetes	28 229	10 046	967	8523	5882	2811
%	14.16%	17.22%	7.02%	14.32%	14.45%	10.42%
Myocardial infarction	25 293	8384	779	8164	4749	3217
%	12.69%	14.37%	5.65%	13.72%	11.67%	11.93%
Stroke	22 318	6551	423	8419	2763	4162
%	11.20%	11.23%	3.07%	14.15%	6.79%	15.43%
Heart failure	27 169	7407	666	10 150	3425	5521
%	13.63%	12.70%	4.83%	17.06%	8.41%	20.47%
Anxiety	4323	1120	553	1189	907	554
%	2.17%	1.92%	4.01%	2.00%	2.23%	2.05%
Depression	29 717	8457	2492	8209	7214	3345
%	14.91%	14.50%	18.08%	13.79%	17.72%	12.40%
Bipolar disorder and other psychosis	1180	315	169	248	321	127
%	0.59%	0.54%	1.23%	0.42%	0.79%	0.47%
Medication						
Beta-blockers	49 222	16 605	2239	14 875	10 731	4772
%	24.70%	28.46%	16.24%	25.00%	26.36%	17.69%
Glycosides	16 800	5258	346	6121	2352	2723
%	8.43%	9.01%	2.51%	10.29%	5.78%	10.10%
Class I and III anti-arrhythmics	8432	2854	642	2252	2057	627
%	4.23%	4.89%	4.66%	3.78%	5.05%	2.32%
Calcium channel blockers	46 405	15 909	994	15 503	8544	5455
%	23.28%	27.27%	7.21%	26.05%	20.99%	20.23%
Oral anti-coagulants	14 072	5355	666	3944	3227	880
%	7.06%	9.18%	4.83%	6.63%	7.93%	3.26%

Clusters 3 and 5 had more female representation. Age was an important feature for the clustering of individuals, with one cluster comprising young individuals aged <50, a second cluster including mainly middle-aged individuals aged between 50 and 70, and the three remaining clusters were made of elderly individuals aged >70.

**Figure 2:**
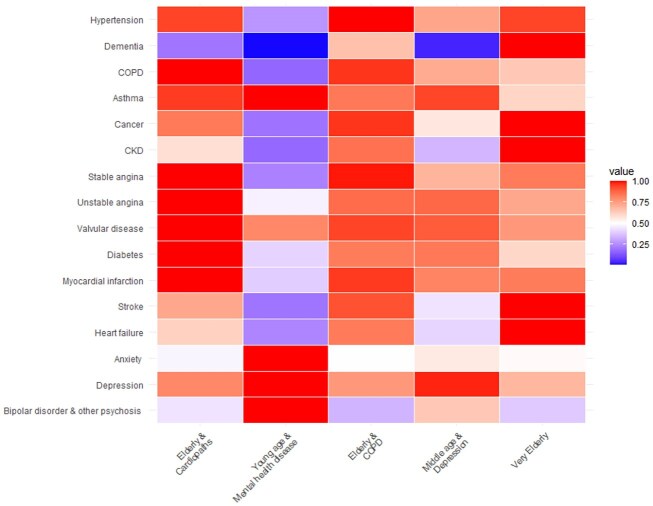
Heat-map with relative prevalence of risk factors across clusters. Each cell represents the relative prevalence of a given risk factors compared with the cluster with higher prevalence. All comparisons vs. the cluster with higher prevalence had a *P*-value < 0.05 (χ^2^ test), except for stable angina (Cluster 1 vs. 3), and depression (Cluster 2 vs. 4).

In Cluster 1, ‘*elderly and cardiopaths’,* all patients were over 70 years old, and the gender distribution was almost equal. The high relative prevalence rates indicated that the patients in this cluster had significantly higher prevalence of numerous co-morbidities at the time of AF diagnosis, namely hypertension, stable angina, unstable angina, myocardial infarction, valvular disease, and diabetes after scaling (*Figure 2*).

In Cluster 2, ‘*young age and mental health disease’,* there was a male predominance (nearly 70%) and nearly all subjects were aged from 18 to 50. This was the cluster with highest prevalence of depression (nearly 20%) and asthma (nearly 15%), and with higher mean BMI (30.5 kg/m^2^). The highest prevalence rate was observed for anxiety, bipolar disorder, and other psychosis diseases (nearly twice more prevalent than in most other clusters). Low prevalence of cardiac disease and cardiovascular risk factors were observed for this cluster.

All cases in Cluster 3, ‘*elderly and hypertensive’,* were above the age of 70, and females were more represented (nearly 60%). The relative prevalence rate of COPD, hypertension and cancer was as high.

Cluster 4, ‘*middle age and depression’,* was composed of individuals aged between 50 and 70, and two-thirds were men. A high prevalence of depression, ∼20%, was observed. Prevalence of cardiovascular disorders and risk factors was lower than for more advanced age clusters.

In Cluster 5, ‘*very elderly’*, mean age was 92, and all subjects were aged over 70. Women predominated in this cluster (two-thirds or more). The relative prevalence rates were higher for cancer and CKD (both occurring in one quarter of patients), heart failure (20%), stroke (15%), and dementia (9%) (*Figure 2*).

**Figure 3:**
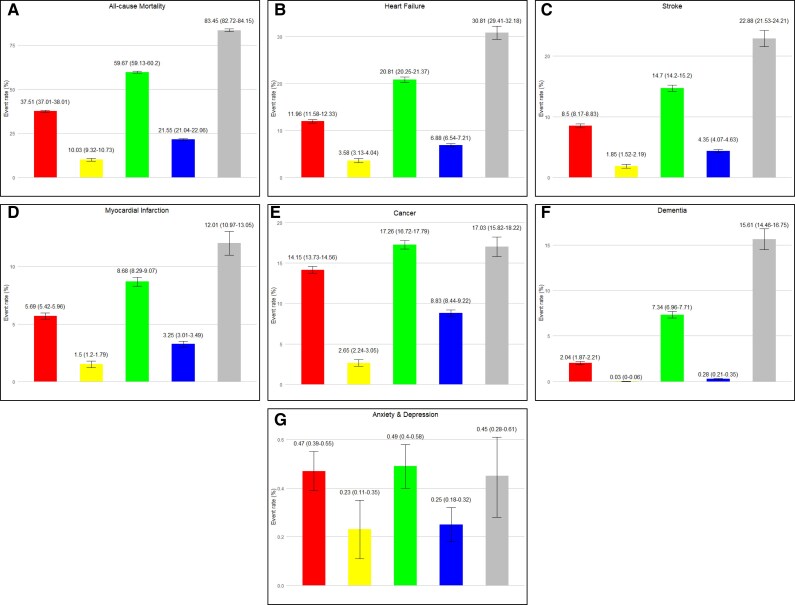
Event rates at 5-years across different clusters.

**Figure 4:**
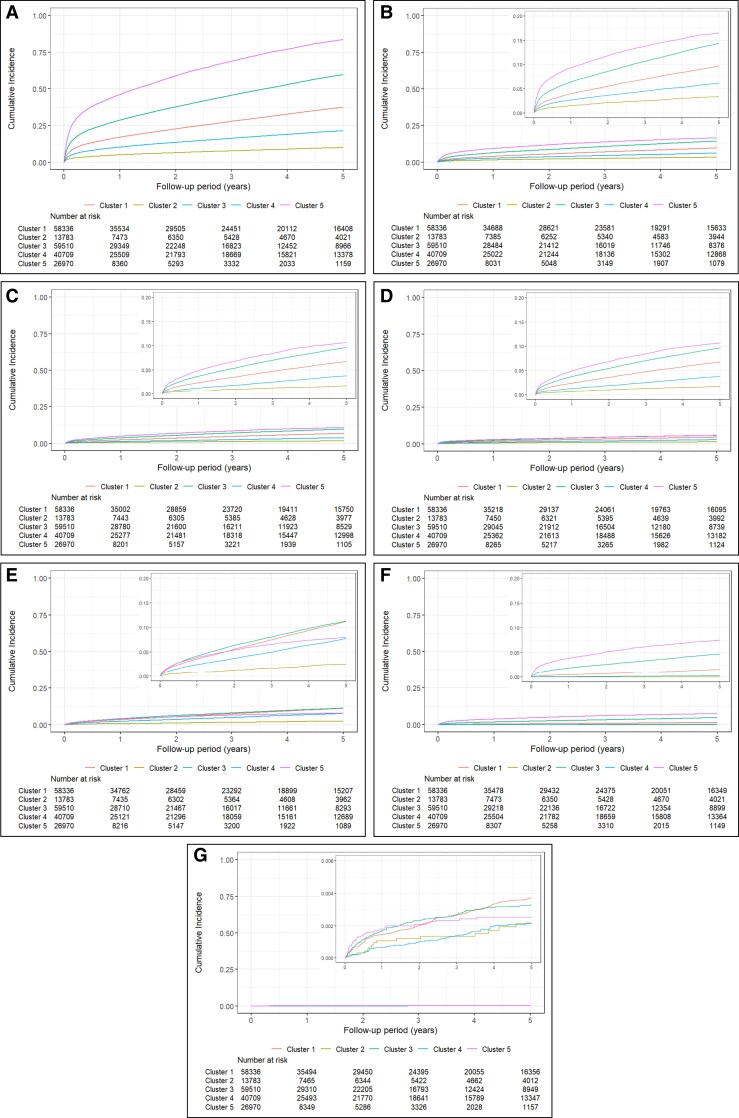
Kaplan–Meier curves for outcomes. (*A*) All-cause mortality and (*B*) heart failure. Custer 1—elderly and cardiopaths; Custer 2—young age and mental health disease; Custer 3—elderly and hypertensive; Custer 4—middle age and depression; Custer 5—very elderly. Panels (*B*), (*C*), (*D*), (*E*), and (*F*) (yy axis 0 to 0.20) and panel (*G*) (yy axis 0 to 0.006) were magnified for better visualization of events across clusters.

Outcomes at 5 years differed across clusters (*Figure 3*). Mortality at 5 years was high, ranging from 10% to over 80% across clusters. Heart failure, stroke, cancer, and myocardial infarction were the main causes for hospitalization (see Supplementary material online, *Table S2*). Anxiety and depression were infrequent causes for hospitalization. Cumulative incidence curves illustrate the differences in time-to-events across clusters (*Figure 4*).

Cluster 1, elderly and cardiopaths, experienced nearly 40% all-cause mortality, nearly 15% cancer hospitalizations, more than 10% heart failure, and 5% myocardial infarction hospitalizations.

Cluster 2, young age and mental health disease, was the healthiest cluster experiencing 10% mortality at 5 years, and the lowest rate of events for all assessed outcomes: 3.6% heart failure, 1.8% stroke, 1.5% myocardial infarction, and 2.6% cancer.

Elderly and hypertensive, Cluster 3, was the second cluster experiencing more events for all outcomes (heart failure in a fifth, stroke in nearly 15% and myocardial infarction in 8.7%), except for cancer and anxiety and/or depression, where elderly and hypertensive were the top cluster at 17.3% and 0.49%.

Cluster 4, middle age and depression, was the second most healthy cluster for all outcomes: cancer and heart failure at 8.8% and 6.8%, respectively, stroke at 4% and myocardial infarction at 3%.

Cluster 5, very elderly, was the sickest cluster, experiencing the highest rate of events, for nearly all outcomes: 83% mortality, 31% heart failure, 23% stroke, 16% dementia, and 12% myocardial infarction. Exceptions were hospitalizations for cancer, where it was comparable to elderly and hypertensive at 17%, and anxiety and/or depression at 0.45%.

**Figure 5:**
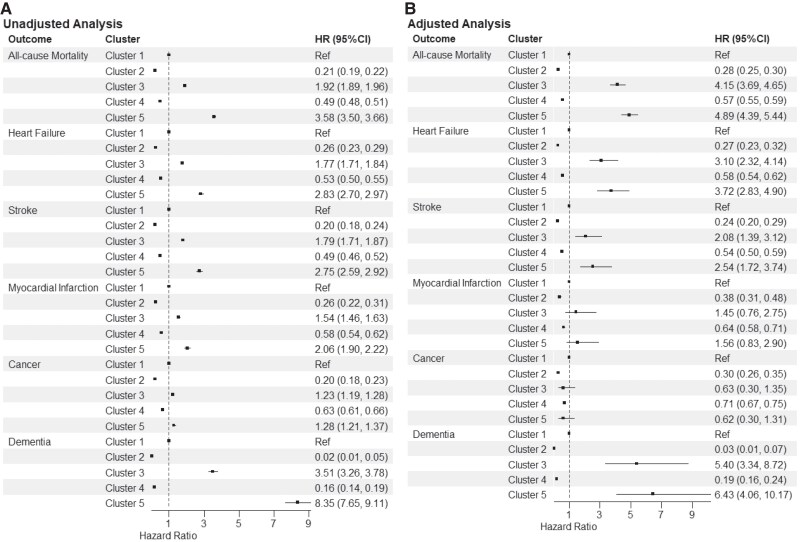
Forest plots of Cox regression results. Custer 1—elderly and cardiopaths; Custer 2—young age and mental health disease; Custer 3—elderly and hypertensive; Custer 4—middle age and depression; Custer 5—very elderly. The number of events of anxiety and depression was too small, resulting in an infinite confidence interval and unstable estimation.

On Cox regression, the clusters were associated with outcomes, even after adjustment for baseline differences (*Figure 5*): compared with Cluster 1, Clusters 2 and 4 (young and middle age patients) were independently associated with a significant reduction of all assessed outcomes; compared with Cluster 1, Clusters 3 and 5 were associated with a significant increase in all-cause mortality, heart failure, stroke and dementia, and no differences were observed for cancer and myocardial infarction.

## Discussion

Utilizing clustering through unsupervised ML, we were able to identify five subphenotypes of AF patients using k-means. With further validation across diverse populations and healthcare settings, these clusters may become clinically actionable, as patients seen in routine clinical practice can be easily assigned to them based on age and presence of readily available clinical variables. The fact that these five AF subphenotypes experienced different outcomes and event rates at 5 years, suggests the possibility of specific tailored therapy and intervention priorities, which requires further investigation. Importantly, unlike previous clustering attempts in the AF population,^24–36^ we included mental health-related features to inform the model and assessed mental health outcomes at 5 years across the identified subphenotypes.

Biological networks often exhibit power-law distributions in their clustering patterns, leading to clusters of varying sizes rather than uniform ones.^37,38^ Therefore, our findings of five clusters of unequal size should not be considered unexpected. Importantly, the clusters were independently associated with the assessed outcomes, suggesting that the baseline differences do not account for the association. Instead, the cluster itself or some unmeasured factors within it may influence the risk of the event occurring.

The model was internally validated using 15-fold cross validation. The identified clusters show resemblances with clusters identified by other authors^24–36^ (*Table 3* and Supplementary material online, Table  *S3*). Saito and colleagues also identified five clusters, with age playing an important role in the process, and similarly to our study identified one cluster of very elderly AF patients with women predominance, and a cluster of younger patients with lower comorbidity burden.^32^ Indeed, Pastori *et al.* identified four clusters, and similarly to our study also identified a cluster of young patients with higher BMI, oldest patients with women, one cluster with middle age and pulmonary disease.^30^ The observed overlap of some clusters identified in our study with clusters identified in other cohorts and countries, utilizing other clustering methods (hierarchical clustering) support the reproducibility of clustering to identify AF clinical subphenotypes, going beyond uninterpretable artificial intelligence constructs.

**Table 3:** Main findings of clustering studies of AF patients in the literature

	Inohara 2018	Ogawa 2021	Proietti 2021	Suzuki 2021	Vitolo 2021	Watanabe 2021	Pastori 2022
*n*	9749	4304	9363	573	3980	7406	5717
Cohort	ORBIT-AF registry, 174 sites	FUSHIMI AF registry, 81 centres	ESC/EHRA EORP-AF registry, 250 centres	Shinken Database, Single centre	AMADEUS and BOREALIS RCTs	J-RHYTHM registry, 158 sites	START registry, 7 sites
Country	USA	Japan	27 countries	Japan	Multinational	Japan	Italy
Age (Mean ± SD)	75 ± 11	73.6 ± 10.9	71 ± 11	80.4 ± 4.3	69.7 ± 8.5	70 ± 10	75.0 ± 9.6
Female	42.6%	40.3%	39.6%	43.5%	36.7%	29.2%	45.3%
ML approach	Hierarchical clustering using the Ward minimum variance method	Hierarchical cluster analysis using Ward’s minimum variance method	Hierarchical cluster analysis based on Ward’s Method and Squared Euclidean Distance	Hierarchical clustering using Ward’s linkage hierarchical algorithm	Hierarchical clustering using the Ward minimum variance method	Hierarchical cluster analysis (Ward’s method)	Model-based clustering
Number of Included Features	60 variables mainly clinical variables, and a few laboratory, ECG and echocardiogram parameters	42 variables Clinical	22 variables Clinical	Risk scores for 4 outcomes obtained through multivariate logistic regression analyses and composed of 22 variables (clinical and laboratory test results)	12 variables Clinical	40 variables Clinical and laboratory test results	12 variables Clinical
Mental Health Features	None	None	None	None	None	None	None
*n* of clusters	4	6	3	3	4	4	4
Cluster description	Low comorbid burden Younger/behavioral disorder (i.e. alcohol, smoking, drugs and liver disease) Resembling patients with tachycardia-bradycardia, with implanted devices With atherosclerotic co-morbidities	Younger age/less co-morbidities Elderly and low on co-morbidities High prevalence of CV risk factors Atherosclerotic co-morbidities History of stroke Very elderly	1: older patients with non-cardiac comorbidities 2: younger patients with few comorbidities 3: older patients with high prevalence of CV risk factors and comorbidities	standard risk High thromboembolic and major bleeding risk high mortality and HF risk	Lowest BMI and less CV risk factors and comorbidities Most likely male, high CV risk and obese Youngest, most likely make, high CV risk and co-morbidities—HF and CAD Older, most likely female, higher burden non-CV co-morbidities	Younger, with lower BMI and less co-morbidities Highest proportion of hypertension History of bleeding Oldest with very high prevalence of CAD and HF	Youngest and low co-morbidities low CV risk and high cancer high CV risk and more men Oldest, more women and more cerebrovascular disease
Anti-coagulants	76%	55.3%	85.6%	81.7%	100%	86.2%	100%
Assessed outcomes	Composite of CV death, MI, stroke/SE, or TIA Individual components All-cause death New-onset HF All-cause hospitalization, CV hospitalization Bleeding hospitalization Major bleeding	Composite of CV mortality, stroke, SE, TIA, or MI. All-cause mortality, major bleeding, and hospitalization for HF	Duration of hospitalization Utilization of health care resources CV events (including stroke, TIA and any SE, any ACS and CV death) All-cause death Composite of CV events and/or all-cause death	All-cause mortality Thromboembolism Major bleeding HF	Stroke or SE MI Cardiovascular death All-cause death Major bleeding	All-cause mortality Thromboembolism Major bleeding	All-cause mortality

	Bisson 2023	Saito 2023	Suzuki 2023	Krittayaphong 2024	Ng 2025	Romiti 2024	Current Study
*n*	3434	6907	2445	3405	2019	32 560	199 308
Cohort	Loire Valley Atrial Fibrillation cohort, 4 sites	SAKURA AF and RAFFINE registries, 117 sites	ANAFIE Registry, 1273 sites	COOL-AF Registry, 27 hospitals	EUROSAF 24 geriatric centres	GLORIA-AF registry	UK Nationwide CPRD dataset
Country	France	Japan	Japan	Thailand	12 European countries	Global	UK
Age (Mean ± SD)	70.3 ± 17	72.1 ± 9.5	82.3 ± 5.5	67.8 ± 11.3	82.9 ± 7.5	70.0 ± 10.5	75.4 ± 12.6
Female	42.8%	29.1%	47.9%	41.8%	57%	45.4%	48.6%
ML approach	hierarchical clustering method using Ward's linkage criterion	K-prototype method	hierarchical clustering using Ward’s linkage hierarchical algorithm	hierarchical clustering using the Ward minimum variance method	Latent class analysis	Latent class analysis	k-means
Number of Included Features	25 variables Clinical	14 variables Clinical and laboratory test results	NA Clinical and laboratory test results	46 variables: Clinical, ECG and echocardiogram	28 variables Broad disease groupings/conditions	18 variables Clinical	25 variables Clinical
Mental Health Features	None	None	None	None	‘Mental and behavioural disorders’	None	Yes
*n* of clusters	3	5	2	3	3	6	5
Cluster description	Younger and low on co-morbidities Old patients with permanent AF, cardiac pathologies and a high burden of CV Old females with high burden of CV co-morbidities	Youngest, highest BMI and lowest rate of IHD Nearly all with hypertension Older patients without hypertension Oldest patients, mainly women, high prevalence HF Older patients with high prevalence of IHD and diabetes	elderly patients with paroxysmal AF and a high proportion of catheter ablation history very elderly patients with a high prevalence of previous major bleeding	1: older adults with a high proportion of CV risk factors and comorbidities; 2: relatively young adults with a small proportion of risk factors or comorbidities 3: mainly older adults with lower prevalence of risk factors and comorbidities than Cluster 1.	Hypertensive, other circulatory conditions, metabolic diseases; Digestive diseases, infection, injury, non-specific clinical and laboratory abnormalities; Heart failure, respiratory diseases	Low complexity CV risk factors Atherosclerotic Thromboembolic Cardiometabolic High complexity	Elderly and cardiopaths young age and mental health disease Elderly and hypertensive Middle age and depression Very elderly
Anti-coagulants	0%	93.2%	0%	75.4%	59%	81.1%	6.6%
Assessed outcomes	Composite of stroke, SE, death and all-cause death Stroke Major bleeding	All-cause mortality Major bleeding Stroke MI	Stroke or SE Major bleeding Intracranial haemorrhage All bleeding Death from CV cause All-cause mortality HF	All-cause mortality Stroke or SE MI HF	All-cause mortality	Composite of all-cause death and major adverse CV events All-cause mortality CV mortality Major adverse CV events Composite of stroke, TIA and SE Major bleeding	All-cause mortality HF MI Stroke Cancer Dementia Anxiety and/or Depression

ORBIT-AF, outcomes registry for better informed treatment of atrial fibrillation; EORP-AF, ESC-EHRA EURObservational Research Programme in AF; CV, cardiovascular; CAD, coronary artery disease; BMI, body mass index; IHD, ischaemic heart disease; ACS, acute coronary syndrome; PAD, peripheral artery disease; HF, heart failure; TIA, transient ischaemic attack; SE, systemic embolism; MI, myocardial infarction; CNS, central nervous system; COPD, chronic obstructive pulmonary disease; EUROSAF, EURopean study of older subjects with atrial fibrillation; GLORIA-AF, global registry on long-term anti-thrombotic treatment in patients with atrial fibrillation; CPRD, clinical practice research datalink.

Most of the studies, we identified in the literature utilized hierarchical clustering,^24–29,31,33,34^ and two used latent class analysis.^35,36^ In our dataset, k-means performed better. K-means is generally faster and more scalable than hierarchical clustering, especially with large datasets.^39^ Furthermore, the better results suggests that the dataset follows the k-means assumptions more closely: grouping is done by minimizing the sum of the squares of distances between the data and the corresponding cluster centroid, making it effective for spherical or well-separated clusters^40^; K-means is suitable for handling large datasets with variability, whereas hierarchical clustering can be sensitive to noise, and K-medoids may be slower due to its reliance on actual data points as medoids.^39^ While K-means can be sensitive to outliers, K-medoids is more robust. However, in datasets without significant outliers, K-means often outperforms K-medoids in terms of efficiency and clustering accuracy.^41^

Nearly a fifth of AF patients had mental health disorders (depression, anxiety, bipolar disorder, and other psychosis), with over-representation in two clusters. Depression was slightly more prevalent—20% to 30% higher—among younger and middle-aged AF patients. The differences in baseline prevalence of psychiatric disorders were most pronounced for bipolar disorder, psychosis, and anxiety. Although anxiety had a relatively low prevalence, it was a striking feature of the younger cluster, where it was at least twice as frequent and affected 1 in every 20 individuals. During the 5-years of follow-up 2% to 3% of patients required hospitalization for these conditions. Although this was much lower than the prevalence observed for outcomes like cardiovascular disorders—the primary driver of hospitalizations—it underscores the importance of mental health for a subset of patients. This finding reinforces the need to prioritize mental health management and improvement as part of a comprehensive, integrated, and holistic approach to AF care.^42,43^ To the best of our knowledge, this is the first study attempting to subphenotype the AF population resorting to clustering and placing an emphasis on mental health disorders.

Mental health disorders have been associated with worse outcomes in patients with AF.^44,45^ Cognitive behavioural therapy has been shown to improve health-related quality of life and reduce psychological distress in AF patients.^46^ Additionally, psychotherapy and/or anti-depressant prescriptions may reduce hospitalizations and mortality in cardiovascular conditions.^47^ While the original integrated ABC pathway publication acknowledges the psychological morbidity of AF patients,^42^ it has been suggested that the current AF management often overlooks key aspects such as psychosocial well-being, mental health, and conditions like anxiety and depression.^48^ Brandes *et al*. have recently advocated for integrating psychologists into the AF care team and extending the care pathway to ‘ABCD’ and treating patients’ underlying mental health issues, including depression.^48^

The identified clusters suggest an important association of AF with life trajectory, with age being important in their definition. It is therefore, hypothesized that as AF patients age, they may migrate or evolve between clusters, and experience changes in their comorbidity profile and potential complications they are at risk.

Our study has some strengths that deserve to be emphasized. First, the discovery power in our cohort surpasses all previously published literature, with the size of our AF population being 10 times larger than all other studies combined (*Table 3*). Second, we tested three clustering methods and chose the one that performed better. Also, five previous publications utilized hierarchical clustering, which was the worst performing approach in our cohort, and hence was not utilized. Finally, we assessed a broader spectrum of outcomes at 5 years, including standard cardiovascular outcomes, and also assessing cancer, dementia and anxiety or depression.

Further improvement of this model with a more thorough characterization of the AF population should be pursued. Further attempts may need to consider other feature categories, namely cardiac imaging, ECG, genetics or other biomarkers. A previous publication by Donoso *et al*. utilized surface ECG signal to categorize AF patients into five clusters,^49^ suggesting such features are of importance for subphenotyping, and some of the studies we identified have utilized blood test results,^25,29,32,33^ as well as ECG or echocardiographic features.^24,34^

The labels assigned to our clusters are based on the over-representation, and higher relative prevalence, using the co-morbidities that peak in each cluster, and demographic data to assign a label. Hence, misinterpretation needs to be avoided as, despite having one cluster labelled as ‘elderly and cardiopaths’, ischaemic heart disease and heart failure are also frequently seen in other clusters (albeit with lower relative prevalence). Furthermore, cluster labelling primarily depends on dominant co-morbidities and may oversimplify the underlying pathophysiological mechanisms or miss subtle nuances and dynamic behaviour not captured within the rigid and static cluster categories.^50^

### Limitations

Some limitations in this investigation need to be emphasized. First, the nationwide dataset is limited to the UK population and may not be representative of other countries or regions. Second, external validation of our findings, which is essential for assessing the consistency and reproducibility of these clusters, is currently lacking. However, as mentioned above, we observed similarities between some of the clusters we identified and those reported in previous publications (*Table 3*). Third, as for every observational analysis, there is the risk of unmeasured risk factors or co-morbidities, with cluster formation being dependent on data granularity and quality. We overcame this limitation by including in study key prognosis factors supported by the previous literature relating to AF. Fourth, due to the nature of the data we had no access to information on AF type (paroxysmal, persistent, or permanent). However, as previously stated, the clinical utility of such a temporal pattern/episode-based classification remains inconclusive.^9^ Furthermore, in other clustering studies where AF type was included as a feature in the model, this did not appear as one of main contributors to the different clusters.

Finally, the number of patients on anti-coagulants was low. This is explained by the fact that use of anti-coagulants was assessed at the time of AF diagnosis and some patients had not yet been started on these agents at that time.

## Conclusion

Using unsupervised clustering we identified 5 distinct AF subphenotypes with different prognosis. The clusters were labelled based on the most prevalent demographic features and relative prevalent co-morbidities. Management of mental health should be part of the integrated health care in this population, as mental health disorders are prevalent across all clusters, with slightly higher involvement in two. Further studies are required on impact of cluster definition for tailoring AF-specific treatment.

## Supplementary Material

ztaf032_Supplementary_Data

## Data Availability

Data for this study were provided by United Kingdom’s Medicines and Healthcare products Regulatory Agency following approval by the Independent Scientific Advisory Committee (17_205), and can be made available to other researchers following application via the Clinical Practice Research Datalink (CPRD) website (https://www.cprd.com/).
