# Supplementary material for: Clustering of electronic health records in atrial fibrillation patients and impact on prognosis and patient trajectories: a UK linked-dataset study
Source: Eur Heart J Digit Health. 2025 Apr 5;6(4):797–810. doi: 10.1093/ehjdh/ztaf032 (PMC12282383; doi:10.1093/ehjdh/ztaf032)
Supplement: ztaf032_Supplementary_Data [file ztaf032_supplementary_data.docx]

**Supplementary Material**

**Table S-1.** Definitions of risk factors included as baseline covariates in cluster analysis

| **Demographic variables** | **Type** | **Baseline measurement** | **Definitions** |
| --- | --- | --- | --- |
| Gender | Binary: male / female |  |  |
| Age at diagnosis with AF | Continuous | >=18 |  |
| Smoking status | Binary:   Smokers/Non-smokers |  | https://caliberresearch.org/portal/show/smoking_status_gprdhttps://caliberresearch.org/portal/show/smoking_status_gprd (categories 2,3,4) |
|  |  |  | ICD10: F17 |
| Alcohol | Binary: Drinkers/Non-drinkers |  |  |
| **Comorbidities** | **Type** | **Baseline measurement** | **Definitions** |
| Diabetes | Binary: Yes/No | Until the development of AF | https://www.caliberresearch.org/portal/show/dm_gprd (categories 3,4,6) |
|  |  |  | https://www.caliberresearch.org/portal/show/dm_hes (categories 3,4,6) |
| Hypertension | Binary, based on hypertension codes: Yes / no | Until the development of AF | https://www.caliberresearch.org/portal/show/ht_gprd (categories 3,4) |
|  |  |  | https://www.caliberresearch.org/portal/show/ht_hes (categories 3,4) |
| Stable angina | Binary: Yes / no | Until the development of AF | https://www.caliberresearch.org/portal/show/sa_diagnosis_gprd (category 4) |
|  |  |  | https://www.caliberresearch.org/portal/show/angina_heshttps://www.caliberresearch.org/portal/show/angina_hes |
| Unstable angina | Binary: Yes / no | Until the development of AF | https://www.caliberresearch.org/portal/show/unangina_gprd (category 3) |
|  |  |  | ICD10: I20.0, I24.0, I24.8, I24.9 |
| Myocardial infarction | Binary: Yes / no | Until the development of AF | https://www.caliberresearch.org/portal/show/myo_infarct_gprdhttps://www.caliberresearch.org/portal/show/myo_infarct_gprd (categories 3,4,5) |
|  |  |  | ICD10: I21 |
| Stroke | Binary: Yes / no | Until the development of AF | https://www.caliberresearch.org/portal/show/ischaemic_stroke_gprd(category 3) |
|  |  |  | https://www.caliberresearch.org/portal/show/haem_stroke_gprd (categories 3-8) |
|  |  |  | https://www.caliberresearch.org/portal/show/stroke_nos_gprd (category 3) |
|  |  |  | ICD10: I60, I61, I63, I64, I62.0, I62.1, I62.9, G46.3, G46.4, G46.5, G46.6, G46.7, |
| Dementia | Binary: Yes / no | Until the development of AF | https://www.caliberresearch.org/portal/show/dementia_gprdhttps://www.caliberresearch.org/portal/show/dementia_hes (categories 2-5) |
|  |  |  | https://www.caliberresearch.org/portal/show/dementia_heshttps://www.caliberresearch.org/portal/show/dementia_gprd (categories 2-5) |
| Heart failure | Binary: Yes / no | Until the development of AF | https://www.caliberresearch.org/portal/show/hf_gprd (categories 3,4,5,6) |
|  |  |  | https://www.caliberresearch.org/portal/show/hf_hes |
| Chronic obstructive pulmonary disease | Binary: Yes / no | Until the development of AF | https://www.caliberresearch.org/portal/show/copd_gprdhttps://www.caliberresearch.org/portal/show/copd_gprd (categories 3,5)  https://www.caliberresearch.org/portal/show/copd_hes (categories 3,5) |
| Chronic kidney disease | Binary: Yes / no | Until the development of AF | https://www.caliberresearch.org/portal/show/renal_gprd (categories 3-7)  https://www.caliberresearch.org/portal/show/renal_hes (categories 3-7) |
| Cancer | Binary: Yes / no | Until the development of AF | https://www.caliberresearch.org/portal/show/cancer_gprd |
|  |  |  | https://www.caliberresearch.org/portal/show/cancer_hes |
| Asthma | Binary: Yes / no | Until the development of AF | ICD10 J45, J46 and corresponding Read code |
| Valvular disease | Binary: Yes / no | Until the development of AF | ICD10: I05. I06. I07, I08, I34, I35, I36, I37 and corresponding Read code. |
| Depression | Binary: Yes / no | Until the development of AF | ICD 10: F32, F33, F34, F38 and corresponding Read code |
| Anxiety | Binary: Yes / no | Until the development of AF | ICD 10: F32, F33, F34, F38 and corresponding Read code |
| Bipolar disorder and other psychosis diseases | Binary: Yes / no | Until the development of AF | ICD 10: F32, F33, F34, F38 and corresponding Read code |
| **Prescribed Medication** |  |  | **Definitions** |
| Glycosides | Binary: Yes / no | one year before and after the development of AF | BNF Chapter 2.1 |
| Antiarrhythmics | Binary: Yes / no | one year before and after the development of AF | BNF Chapter 2.3 |
| Beta-blockers | Binary: Yes / no | one year before and after the development of AF | BNF Chapter 2.4 |
| Calcium channel blockers | Binary: Yes / no | one year before and after the development of AF | BNF Chapter 2.6.2 |
| Oral anticoagulants | Binary: Yes / no | one year before and after the development of AF | BNF Chapter 2.8.2 |

| **Table S-2.** All-cause mortality and hospitalizations at 5 years |
| --- |

|  | **Elderly & Cardiopaths** | **Young age & Mental health disease** | **Elderly & Hypertensive** | **Middle age & Depression** | **Very Elderly** |
| --- | --- | --- | --- | --- | --- |
| All-cause Mortality (%) | 37.51  (37.01-38.01) | 10.03  (9.32-10.73) | 59.67  (59.13-60.2) | 21.55  (21.04-22.06) | 83.45  (82.72-84.15) |
| Heart Failure (%) | 11.96  (11.58-12.33) | 3.58  (3.13-4.04) | 20.81  (20.25-21.37) | 6.88  (6.54-7.21) | 30.81  (29.41-32.18) |
| Stroke (%) | 8.50  (8.17-8.83) | 1.85  (1.52-2.19) | 14.7  (14.2-15.2) | 4.35  (4.07-4.63) | 22.88  (21.53-24.21) |
| Myocardial infarction (%) | 5.69  (5.42-5.96) | 1.50  (1.20-1.79) | 8.68  (8.29-9.07) | 3.25  (3.01-3.49) | 12.01  (10.97-13.05) |
| Cancer (%) | 14.15  (13.73-14.56) | 2.65  (2.24-3.05) | 17.26  (16.72-17.79) | 8.83  (8.44-9.22) | 17.03  (15.82-18.22) |
| Dementia | 2.04  (1.87-2.21) | 0.03  (0-0.06) | 7.34  (6.96-7.71) | 0.28  (0.21-0.35) | 15.61  (14.46-16.75) |
| Anxiety and/or Depression (%) | 0.47  (0.39-0.55) | 0.23  (0.11-0.35) | 0.49  (0.40-0.58) | 0.25  (0.18-0.32) | 0.45  (0.28-0.61) |

**Table S-3.** Description of features utilized for clustering models in the literature

|  | Inohara 2018 | Ogawa 2021 | Proietti 2021 | Suzuki 2021 | Vitolo 2021 |
| --- | --- | --- | --- | --- | --- |
| Included Features | Age, BMI, Heart rate, diastolic and systolic BP, eGFR, Hematocrit, race/ethnicity, sex, smoking status, cancer, hypertension, osteoporosis, diabetes, hyperthyroidism, hypothyroidism, GI bleed, obstructive sleep apnea, dialysis, hyperlipidemia, anemia, cognitive impairment/dementia, frailty, liver disease, COPD, alcohol abuse, drug abuse, family history of AF, peripheral vascular disease, sinus node dysfunction/sick sinus syndrome, stroke or TIA, congestive HF, significant valvular disease, prior valve replacement/repair, pacemaker, ICD, BiV, CRT-D, history of coronary artery disease, prior MI, prior CABG, any PCI, any DES, prior cardioversion, prior antiarrhythmic drug, catheter ablation of AF, AV node or bundle ablation, sinus rhythm at most recent 12 lead ECG, Intraventricular conduction, ECG evidence of LVH, LVEF, LA enlargement, AF type and symptoms | Age, sex, BMI, pulse rate, diastolic, and systolic BP, smoking, alcohol drinking, prior stroke, type of prior stroke, prior TIA, prior SE, HF, cardiomyopathy, valvular heart disease, hypertension, diabetes, dyslipidaemia, CAD, MI, PAD, CKD, COPD, history of major bleeding, liver dysfunction, haemodialysis, previous valve surgery, device implantation, CABG, PCI, cardioversion, catheter ablation, type of AF, valvular AF, interval of first AF documentation, and symptoms of AF | Age, sex, HF, CAD, valvular disease, hypertension, diabetes mellitus, ischemic stroke, peripheral ischemic events, liver disease, COPD, anaemia, dementia, any cardiomyopathy, hyperthyroidism, hypothyroidism, CKD, obstructive sleep apnoea syndrome, malignancy, and BMI | Age, sex, BMI category, systolic BP category, serum albumin category, hemoglobin category, eGFR category, Charlson’s comorbidity index, fall within 3 years after the initial visit, ischemic heart disease, valvular heart disease, cardiomyopathy, HF, hypertension, dyslipidemia, diabetes mellitus, hyperuricemia, history of ischemic stroke or TIA, history of intracranial hemorrhage, COPD and maintenance dialysis. | Sex, age > 75, anaemia, hypertension, HF, diabetes mellitus, previous stroke/TE, CAD, CKD, type of AF, BMI classes and use of any antiplatelets |
|  | Watanabe 2021 | Pastori 2022 | Bisson 2023 | Saito 2023 | Suzuki 2023 |
|  | Age, height, weight, HR, systolic BP, diastolic BP, CHA_2_DS_2_-VASc, HAS-BLED, hemoglobin, platelets, creatinine, Total cholesterol, TTR of INR, Male sex, AF type, congestive HF, hypertension, age > 75 years, diabetes, previous stroke or TIA, CAD, COPD, cardiomyopathy, malignancy, hepatitis, abnormal renal function, abnormal liver function, alcohol >8U/week, congenital heart disease, hyperthyroidism, Previous bleeding, Class I AADs, Class III AADs, Beta-blocker, calcium channel blocker, digitalis, ACEi or ARB-II type 1 receptor blocker, statin, warfarin, and antiplatelet agent | Age, sex, diabetes, previous cerebrovascular events, previous cardiovascular events, HF, PAD), use of non-vitamin K OAC, cancer, pulmonary disease, smoking habit, and previous major bleeding | Age, sex, permanent AF, hypertension, diabetes mellitus, HF, valve disease, CAD, vascular disease, previous pacemaker or ICD, previous ischemic stroke, previous major bleeding, tobacco smoking, dyslipidaemia, alcohol-related diagnoses, renal insufficiency, dialysis, liver disease, thyroid disease, eGFR, non-steroidal anti-inflammatory drugs, antiplatelet agent, P2Y12 inhibitors and class III AADs | Age, sex, systolic BP, BMI, history of HF, hypertension, diabetes, hemoglobin, platelets, AST, ALT, eGFR, CHA_2_DS_2_-VASc, and HAS-BLED | Continuous variables (predicted values) & categorial variables (predictive probabilities).  Unclear which features. The following were listed on the paper: age, sex, BMI, systolic BP, diastolic BP, HbA1C, eGFR, CHADS2, CHA2DS2VASc, HELT-E2S2, HAS-BLED, history of major bleeding, AF type, non-pharmacological AF therapy, catheter ablation, electrical defibrillation, ICD, pacemaker, HF, MI, Hypertension, diabetes mellitus, CKD, dyslipidaemia and cerebrovascular disease |
|  | Krittayaphong 2024 | Ng 2024 | Romiti 2024 | Current study |  |
| Included Features | Age, BMI, eGFR, hematocrit, diastolic BP, systolic BP, HR, dyslipidemia, CAD, history of MI or unstable angina, history of CABG, history of PCI, history of drug eluting stent, type of AF, pacemaker, NYHA class III/IV, ICD, CRT-P, CRT-D, intraventricular conduction defect, female gender, hypothyroidism, hypertension, diabetes, history of GI bleeding, RRT/kidney transplantation, anemia, dementia, PAD, history of ischemic stroke or TIA, cardioversion history, rhythm control medications, catheter ablation of AF, AV node ablation pacemaker, LBBB, ECG evidence of LVH, current smoker, hyperthyroidism, liver function, alcohol abuse, mild LA enlargement, LA enlargement, severe LA enlargement, mild LVSD, moderate LVSD and severe LV dysfunction | ICD-10 defined 28 broad disease groupings/conditions: certain infectious and parasitic diseases, neoplasms, diseases of the blood and blood-forming organs, diseases of the nervous, respiratory, digestive, musculoskeletal, and genitourinary systems, mental and behavioural disorders, diseases of skin, eye, and ear, congenital malformations, non-specific clinical and laboratory abnormalities, injury, poisoning and certain other consequences of external causes, external causes of morbidity and mortality, and factors influencing health status and contact with health services.  Diabetes mellitus, dyslipidaemia, other endocrine/metabolic diseases, rheumatic heart disease, hypertensive disease, ischemic heart disease, HF/cardiomyopathy/pulmonary hypertension, other forms of heart disease, cerebrovascular diseases, deep vein thrombosis/pulmonary embolism and other circulatory disease | Hypertension, CAD, HF, PAD, history of previous stroke/TIA, history of venous thromboembolism, previous bleeding events, diabetes mellitus, hyperlipidemia, obesity, history of cancer, abnormal kidney function, COPD, emphysema, hyperthyroidism, liver disease, gastrointestinal disease and the presence of neurologic conditions | Gender, age, smoking status, alcohol, comorbidities, diabetes mellitus, hypertension, stable angina, unstable angina, myocardial infarction, stroke, dementia, HF, COPD, CKD, cancer, asthma, valvular disease, depression, anxiety, bipolar disorder and other psychosis diseases, prescribed medication: glycosides, class I and III anti-arrhythmic drugs, beta-blockers, calcium channel blockers, and oral anticoagulants |  |

Legend: CV – cardiovascular; BP – blood pressure; CAD – coronary artery disease; CKD – chronic kidney disease; TE – thromboembolism; CABG - coronary artery bypass surgery; PCI - percutaneous coronary intervention; OAC – oral anticoagulants; BMI – body mass index; IHD – ischemic heart disease; PAD – peripheral artery disease; HF – heart failure; TIA – transient ischemic attack; SE – systemic embolism; MI – myocardial infarction; COPD – chronic obstructive pulmonary disease; LVSD – left ventricular systolic dysfunction; AAD – antiarrhythmic drugs; eGFR – estimated glomerular filtration rate; ICD – implantable cardioverter defibrillator; CRT-P – cardiac resynchronization therapy / biventricular pacemaker; CRT-D – cardiac resynchronization therapy with defibrillator; CABG – coronary artery bypass grafting.

**Figure S-1.** Silhouette plot of one folder resulting from selected clustering methods

k-means


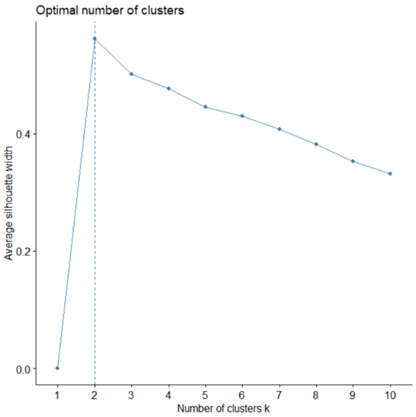

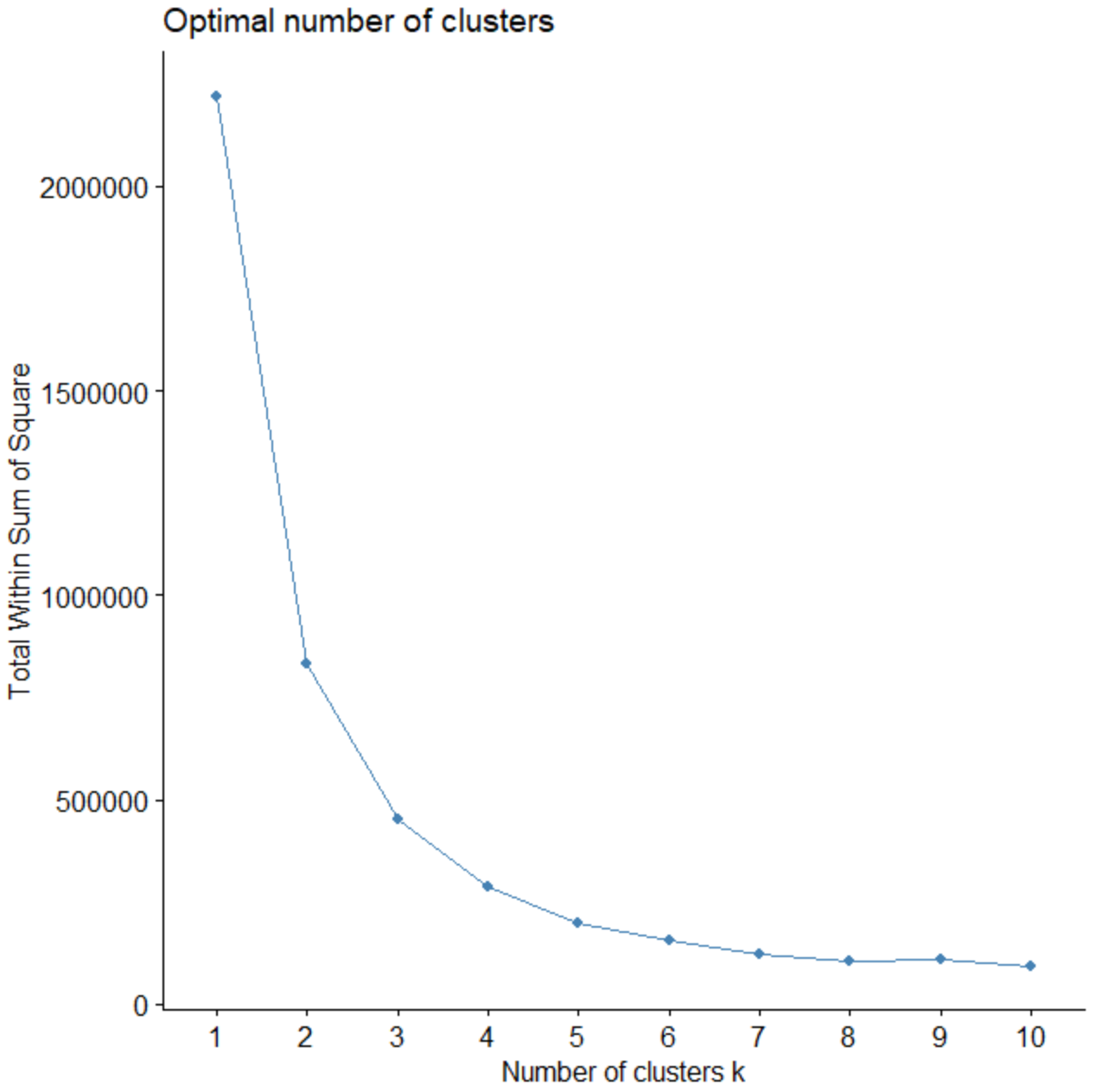


k=5


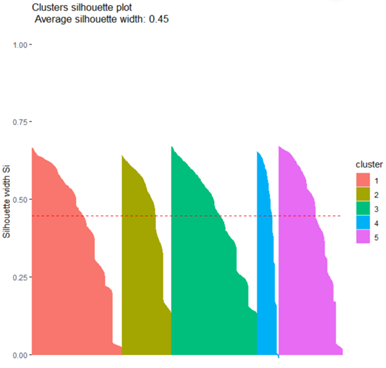


Hierarchical Clustering


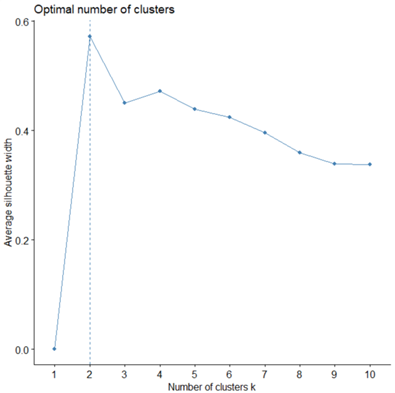

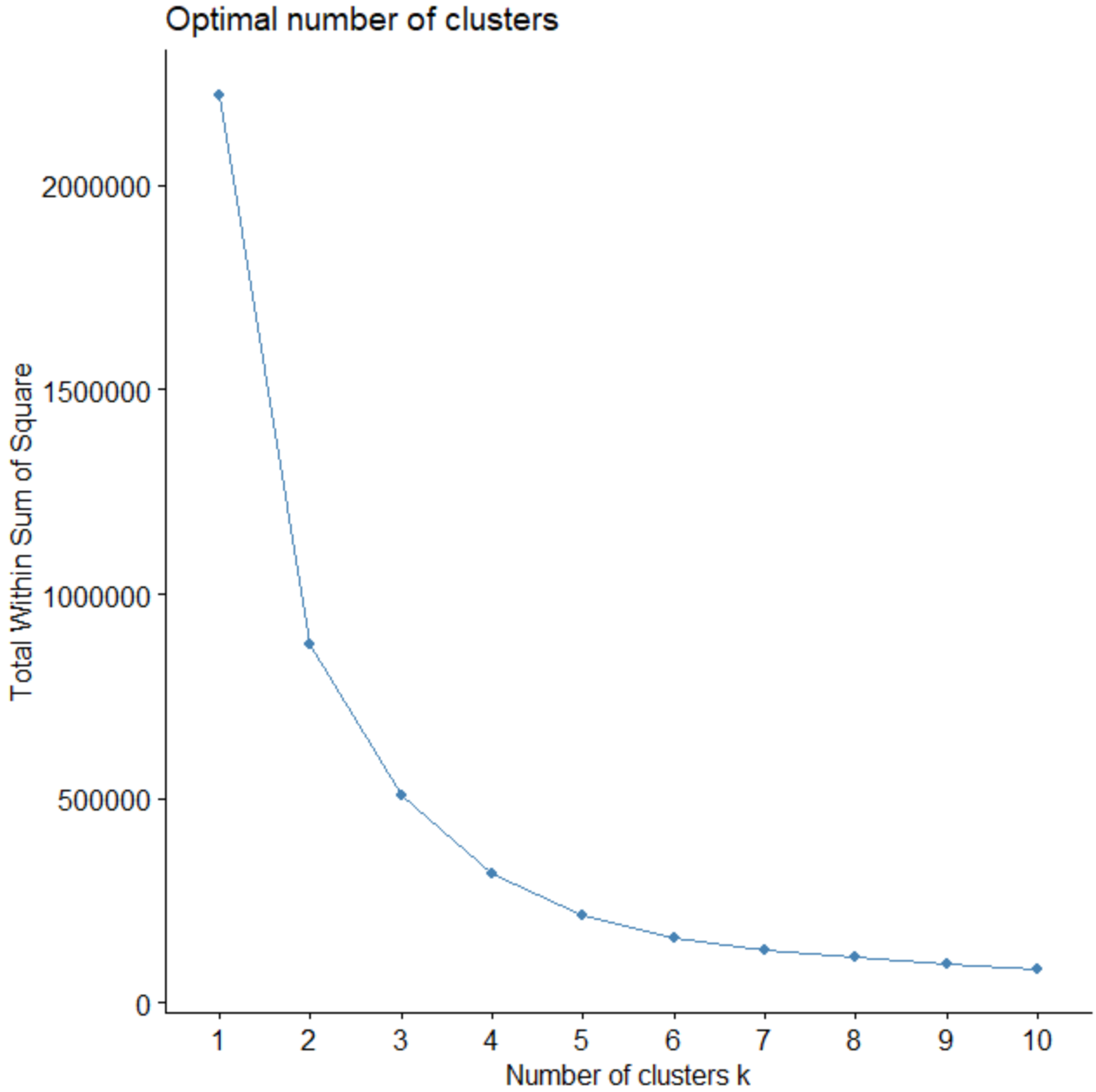


k=5 Dendogram


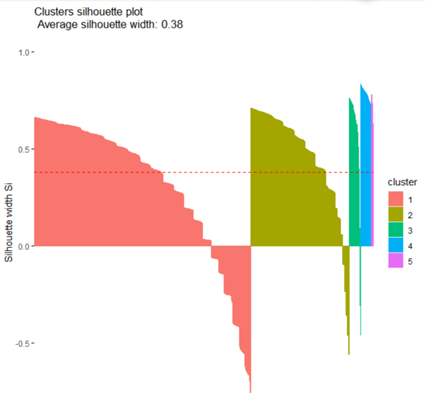

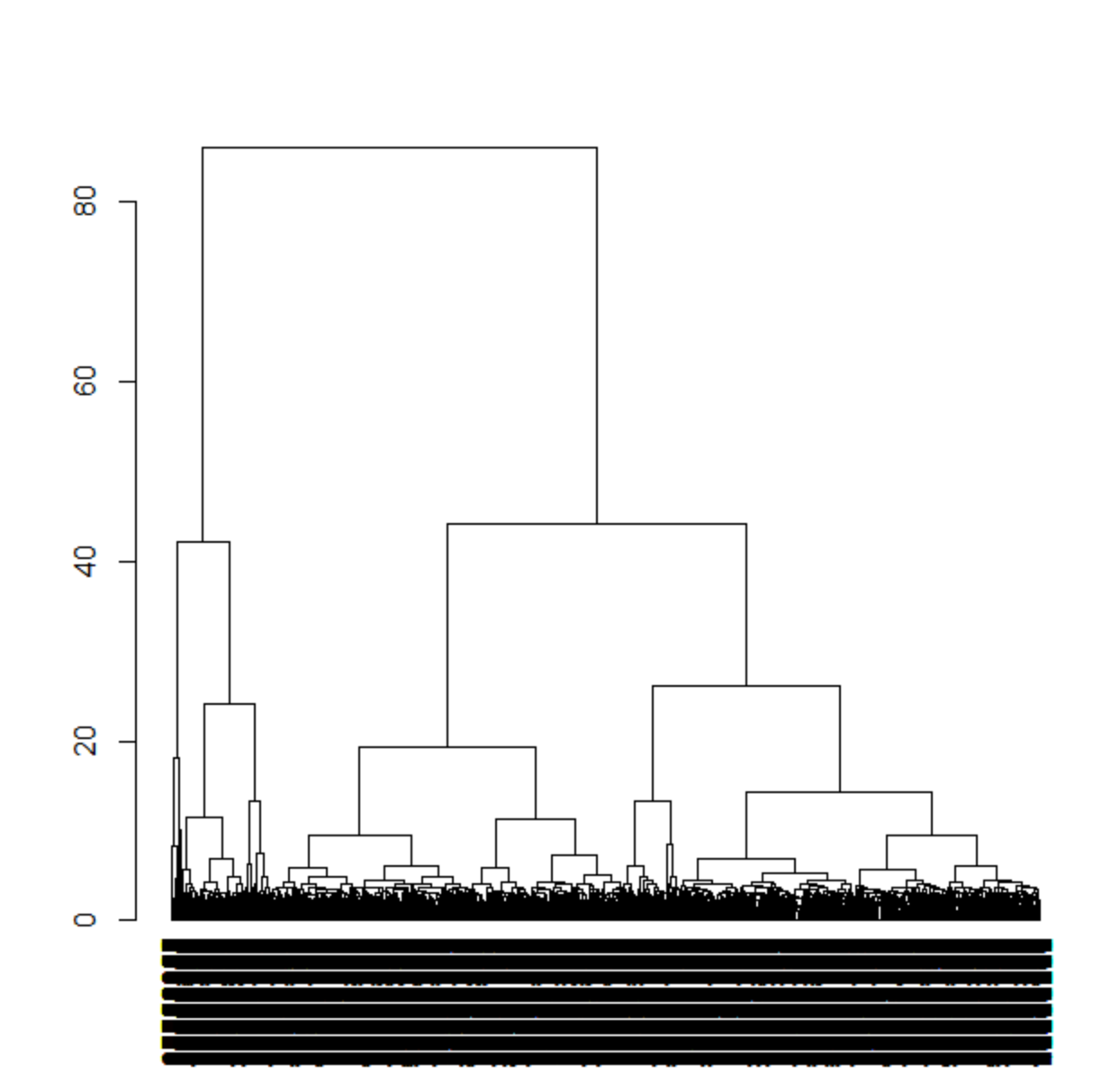


K-Medoids


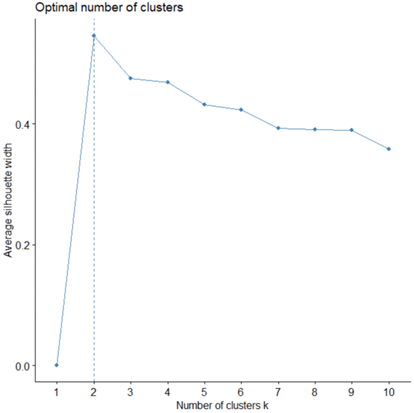

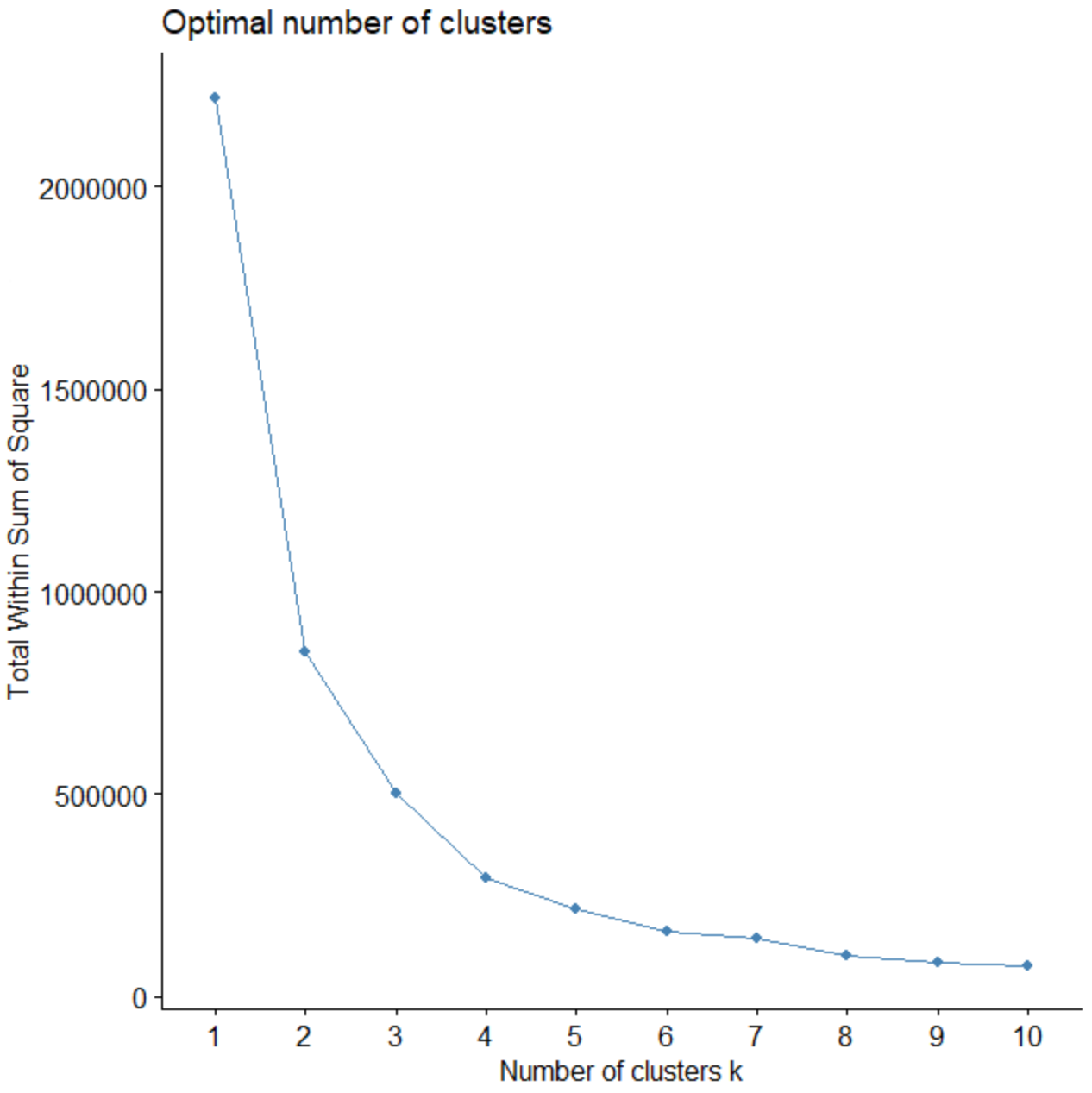


k=5


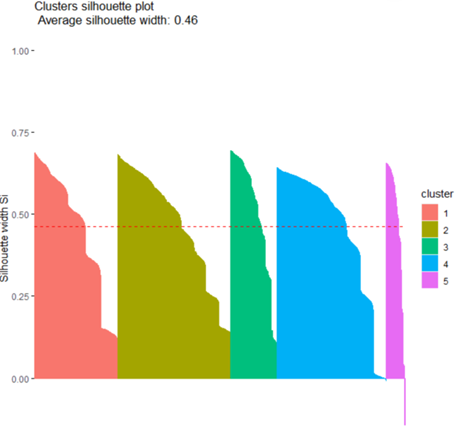


**Figure S-2.** Five-year survival probability to show optimal number of clusters (k = 4-5)

k-means


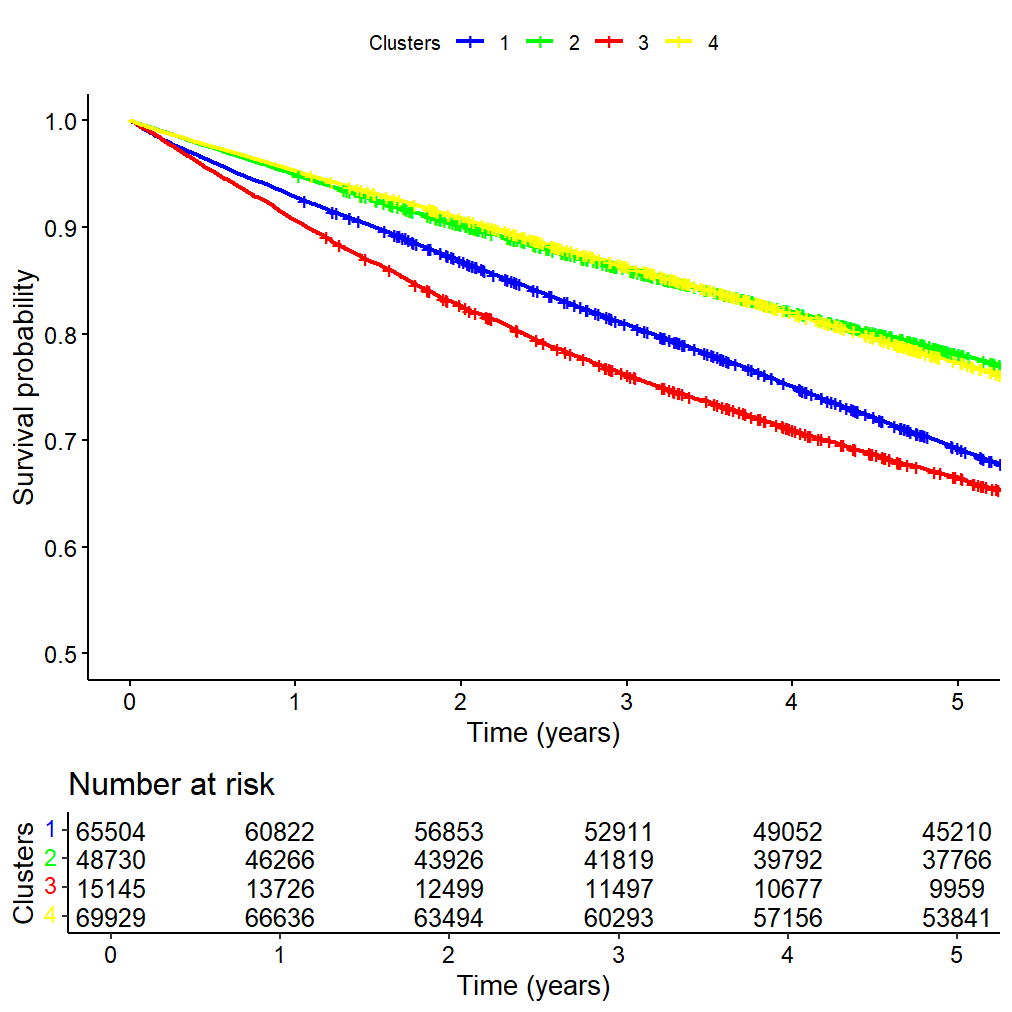

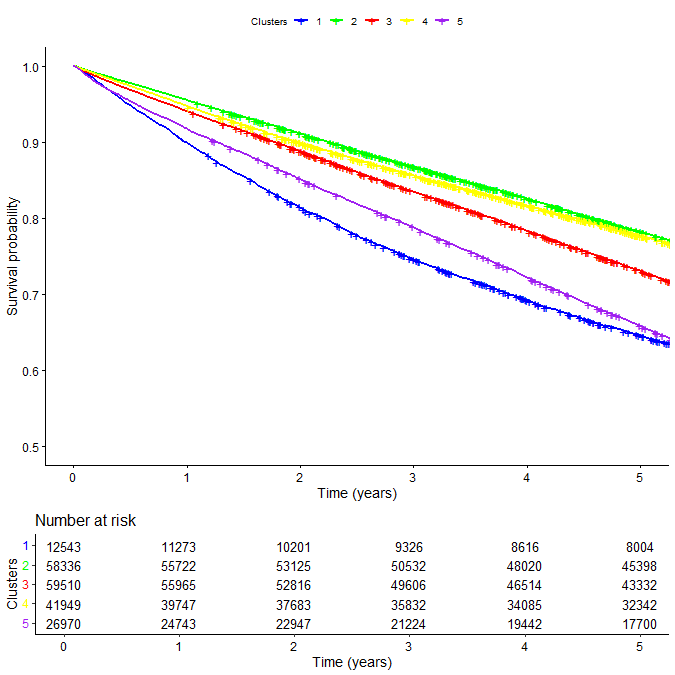


K-medoids


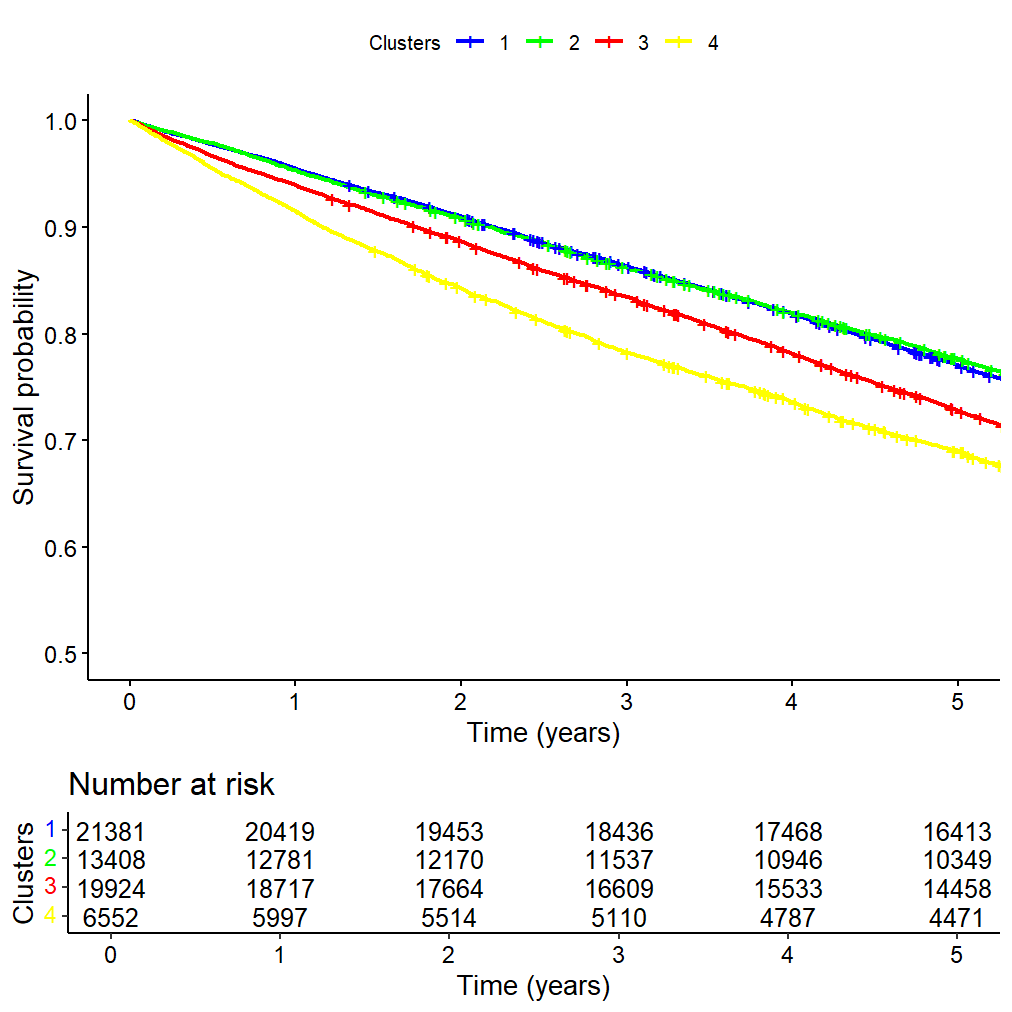

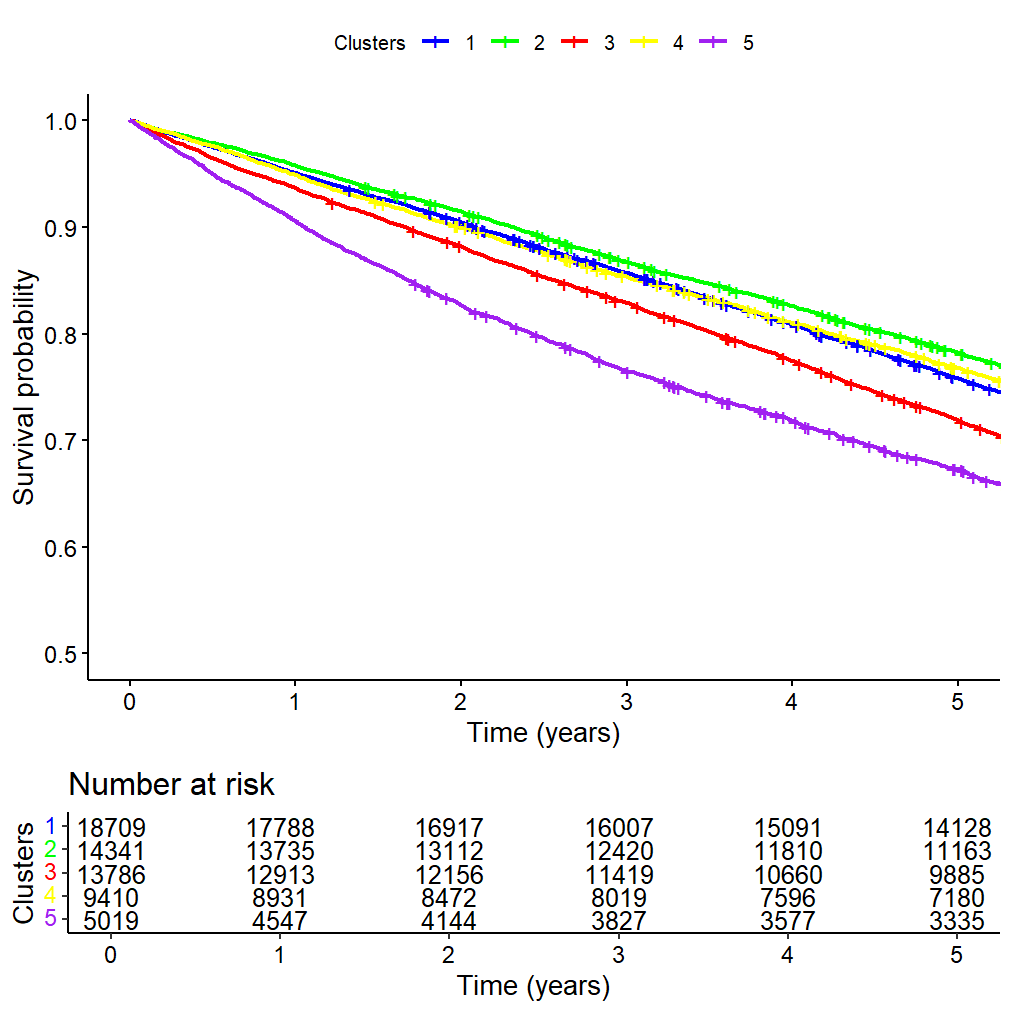


**Figure S-3.** Proportion of patients in each cluster
